# Voluntary Support

**Published:** 1894-02-24

**Authors:** 


### HOSPITAL FINANCE.

### III.—VOLUNTARY SUPPORT.

The very essence of true charity, and hence of the best hospital support, is personal sympathy. State and municipal institutions can hardly fail to exclude this element. Rich endowments, though in most instances starting under the highest motives, tend to fall away from them when deprived of the living influence of the founder. But the continual urgent need of institutions in our midst directed to the relief of suffering daily under our notice, and directly dependent on us for support, constitutes the most forcible of all influences for arousing men to a full recognition of their responsibility one towards another. Who can dare to echo the old cynical excuse, "Am I my brother's keeper?" when he is a daily witness to the thousands who work out an answer to it at the expense of labour, income, and life itself?

Among all nations England is distinguished as having most fully developed the voluntary element in hospital work. In all other countries, and, as has been seen, even in the British colonies, the State or commune lies in wait in the background to supplement, if need be, by forced contribution, the public generosity. In England alone the public is responsible individually instead of collectively; the shame of any deficiency in revenue cannot be shifted on to the shoulders of a party administration, and special liberality can be evoked only as a consequence of the stirring of a real interest in special needs. Anomalies and abuses are not wanting to the system, but while the principle underlying it remains sound its defects can be neither radical nor abiding, but such only as must be met and combated by each generation according to successive development.

It is instructive to examine in detail the amount of money annually contributed to the hospitals and the sources from which this income is derived. In the following statistics the sums derived from invested property, together with patients' payments and probationers' fees, have been deducted from the income of the hospitals, and the result shows the spontaneous offerings, including legacies, towards hospital work in the United Kingdom in 1891:—

| | General. | Special. | Total. |
|---|---|---|---|
| London | £217,000 | £176.880 | £393,880 |
| Provincial | 395,601 | 22,477 | 418,078 |
| Scotch | 124,150 | 17,738 | 141,888 |
| Irish | 20,460 | 3,544 | 24,004 |
| Total | £757,211 | £220,639 | £977,850 |

The voluntary offerings to the hospital amount therefore, it appears, to close upon a million, of which considerably more than one-third is contributed to the hospitals of London alone. This amount does not, however, represent anything like the true extent of the yearly offerings to medical charities. Only the principal institutions have been included, and no calculation has been made for dispensaries, convalescent homes, and other agencies for medical relief.

The chief sources from which these revenues are derived are: (1) Legacies; (2) annual subscriptions; (3) donations; (4) grants from public funds, such as the Hospital Saturday and Sunday Fund. For many years legacies have been a mainstay of the hospitals. Of the total quoted above one-third, or a sum of £298,965, is derived from legacies. For obvious reasons no source of income is more fluctuating than this, but during the last two or three years there has been reason to fear the setting in of a serious decrease under this head. To a greater extent than is at all realised legacies stand to the hospital in the light of mere compensation for definite loss. Those who remember its needs in their wills have commonly been its most generous benefactors during their lifetime; and even as regards money's worth, the change of a lump sum for the yearly subscription is often for the worse, while nothing can atone for the loss of the personal example and influence of the giver. A hospital is not, therefore, necessarily benefited by a good legacy year, which may only mean that death has been busy among its best friends. But a steady and long-continued diminution in legacies, pointing as it undoubtedly would to a diminution of interest in hospital work on the part of the wealthier members of the community, would be a serious calamity, and that not only or chiefly to the charities.

The revenue derived from annual subscriptions amounts to rather over a fourth of what may be called the voluntary income, or a sum of £277,505. The proportion which annual subscriptions bear to the entire revenues of each hospital varies in the most remarkable manner. It is lowest in the London general hospitals, where it amounts to but 10 per cent. In the provincial general hospitals it reaches 24 per cent., and in Scotch special hospitals 36 per cent. The very limited number of annual subscribers to the London hospitals is matter for great regret. It is more than probable that a well-organised system of appeal on the part of the management of these hospitals would result in arousing vast numbers to a sense of their responsibility to the sick poor who are their neighbours. The annoyance of conflicting appeals from various institutions, which tends to bring about a state of indifference to the claims of all, should be avoided by concerted action, and a vigorous effort made to secure the interest of the subscriber in his own hospital by enforcing the value of small and regular contributions. It is believed that many thousands of small subscribers might by this means be enrolled, to whom at present no encouragement in the rendering of regular assistance is given. And it is needless to say that no sounder basis can well be imagined for charitable work than hearty local support. As might be expected, the local feeling is far stronger in the provinces, but it is very unequally exhibited, and the experience of several hospitals where a well-planned attempt to attract subscribers of a guinea and under has been tried, shows that this source of hospital income is exceptionally fertile although practically unutilised.

The question of donations is more complex. Many persons, especially those whose incomes fluctuate largely, refuse to commit themselves to an annual subscription, but will, if applied to, give regularly from time to time what they can afford. This class of donations presents no difficulty beyond that of keeping an accurate register of donors. But by far the larger proportion of the amount annually collected in donations, an amount falling very little short of the revenue from subscriptions, is from such varying and uncertain sources that the problem of collection becomes a very serious one. Of the total amount (£242,330) entered under the head of donations, £4,665 was collected in boxes, presumably in very small sums. No information is forthcoming with regard to the remainder, as to modes of collection, but the various hospitals show so marked a difference in the amounts contributed in donations as to suggest a wide variation in the method adopted for attracting them.

Many persons consider that in this question of collecting money to carry on good and necessary work almost any means are justified by success. There are, however, two serious blunders to be avoided, both of which tend seriously to cripple the benefit accruing to the institution from the donations obtained. The first is the method only too familiar to everyone of obtaining money by a process of worrying. This methods rests on the assumption that people do not like to give, but that they can be forced into it if they are made sufficiently uncomfortable. Brass bands and the hideous rattle of copper coins shaken in boxes are brought into requisition, accompanied by all the rabble of the neighbourhood, and if the result is to any extent satisfactory, from a financial point of view, it is gained at the expense of a serious loss of dignity to the institution, and a lessening of the ties of sympathy which should bind the neighbourhood to it. Collecting cards entrusted to children and to irresponsible persons partake largely, too, of this worrying element. The fact is that English people delight in giving. The whole story of our charities proclaims this aloud, but at the same time they do object very strongly indeed to perpetual small demands, and those who give most generously object most strongly. Moreover, the public is being slowly trained to recognise the responsibility which giving entails. Those who appeal for money should encourage this awakening sense of responsibility (first) by asking with dignity through qualified agents, and (second) by laying before the donors the fullest information possible about the work for which assistance is asked. As to those who never give unless from whim, or to escape importunity, they may be disregarded. Money so offered can be of no moral benefit to the givers, and the glad, spontaneous offerings of the majority, wisely collected, should be sufficient without their extorted aid.

The second error alluded to above is that of spending on the collection an undue proportion of the proceeds. This is very much a matter of experience and for careful adjustment of means to an end. But certain notoriously expensive expedients, such as festival dinners, must always be regarded as very doubtful advantages to the institution it is proposed to benefit. A liberal expenditure in diffusing information in the right quarters about the work and needs of the hospital may be the truest economy, and bear fruit long after the immediate occasion has passed away.

